# Enhanced Regeneration and Hepatoprotective Effects of Interleukin 22 Fusion Protein on a Predamaged Liver Undergoing Partial Hepatectomy

**DOI:** 10.1155/2018/5241526

**Published:** 2018-10-31

**Authors:** Heng Zhou, Guomin Xie, Yudi Mao, Ke Zhou, Ruixue Ren, Qihong Zhao, Hua Wang, Shi Yin

**Affiliations:** ^1^Department of Geriatrics, Anhui Provincial Hospital, Anhui Medical University, Hefei 230001, China; ^2^School of Pharmacy, Anhui Medical University, Hefei 230032, China; ^3^Institute for Liver Disease, Anhui Medical University, Hefei 230032, China; ^4^Department of Oncology, The First Affiliated Hospital of Anhui Medical University, Hefei 230032, China; ^5^Department of Food and Nutrition Hygiene, School of Public Health, Anhui Medical University, Hefei 230032, China

## Abstract

Liver ischemia-reperfusion injury (IRI) and regeneration deficiency are two major challenges for surgery patients with chronic liver disease. As a survival factor for hepatocytes, interleukin 22 (IL-22) plays an important role in hepatoprotection and the promotion of regeneration after hepatectomy. In this study, we aim to investigate the roles of an interleukin 22 fusion protein (IL-22-FP) in mice with a predamaged liver after a two-third partial hepatectomy (PHx). Predamaged livers in mice were induced by concanavalin A (ConA)/carbon tetrachloride (CCl4) following PHx with or without IL-22-FP treatment. A hepatic IRI mouse model was also used to determine the hepatoprotective effects of IL-22-FP. In the ConA/CCl4 model, IL-22-FP treatment alleviated liver injury and accelerated hepatocyte proliferation. Administration of IL-22-FP activated the hepatic signal transducer and activator of transcription 3 (STAT3) and upregulated the expression of many mitogenic proteins. IL-22-FP treatment prior to IRI effectively reduced liver damage through decreased aminotransferase and improved liver histology. In conclusion, IL-22-FP promotes liver regeneration in mice with predamaged livers following PHx and alleviates IRI-induced liver injury. Our study suggests that IL-22-FP may represent a promising therapeutic drug against regeneration deficiency and liver IRI in patients who have undergone PHx.

## 1. Introduction

IL-22 is an emerging CD4+ Th cytokine produced by activated T cells, such as T helper 22 (Th22) cells, Th17 cells, and NK cells [1–4]. As a member of the IL-10 cytokine family, IL-22 plays a role in a variety of tissues and organs by binding to the receptors IL-10R2 and IL-22R1; IL-10R2 is expressed in many types of cells, but the expression of IL-22R1 is limited to epithelial cells in the skin, pancreas, liver, gut, and lung [5–8].

IL-22 plays an important role in protection against damage, strengthening of innate immunity and enhancement of regeneration [8–10]. IL-22 plays important roles in various human and animal liver diseases, such as acute liver injury, viral hepatitis, liver fibrosis, hepatocellular carcinoma (HCC), and alcoholic liver disease [11–19]. Studies from professor Bin Gao have shown that IL-22 is released when T cells are activated and then protects against liver damage caused by a variety of toxins, such as ConA or CCl4, via activation of the STAT3 pathway [20, 21]. IL-22 has been shown to be a significant mediator of the inflammatory response caused by HBV and HCV. In HBV patients, hepatic expression of IL-22 was elevated compared with healthy individuals and the degree of increase was related to the grade of inflammation [22–26]. Compared with normal controls, the expression of numerous genes associated with the IL-22 pathway was significantly upregulated in HBV-infected liver tissues [24]. IL-22 ameliorates liver fibrogenesis by inducing hepatic stellate cell senescence, and it stimulates liver cancer development through activation of the STAT3 pathway [16, 17, 19].

Liver regeneration is a complex process and is closely related to a wide variety of cytokines, hormones, and growth factors [27]. It is generally accepted that serum levels of lipopolysaccharide (LPS) will be upregulated after partial hepatectomy (PHx), which stimulates the Kupffer cells to produce IL-6 and tumor necrosis factor alpha (TNF-*α*). IL-6 targets hepatocytes, triggering activation of the STAT3 pathway, and ultimately promotes hepatocyte survival and proliferation [27–29]. It is an indisputable fact that IL-22 contributes to liver regeneration after PHx. Researchers have found that the serum IL-22 and hepatic IL-22 receptor mRNA levels were significantly upregulated after PHx in mice. Some studies have shown that the promotive properties of IL-22 on liver regeneration after PHx are likely due to interactions with TGF-*α* and IL-6 [30–32]. In addition, IL-22 plays a protective role in liver ischemia-reperfusion injury (IRI) [33]. After treatment with IL-22, the serum aspartate aminotransferase (AST) level decreased but the expression of IL-22R1 in damaged hepatocytes increased, which significantly alleviates IR-triggered hepatocellular damage and decreases IR-related liver inflammation [33]. As mentioned, liver regeneration deficiency and liver injury are still the two major stumbling blocks for patients after PHx, although the liver is a unique organ that has the ability to regenerate after injury or resection. IL-22 has been reported to contribute to liver regeneration after hepatectomy and protect the liver against IRI, and this corresponds to the two problems patients experience after PHx. Therefore, treatment utilizing IL-22 is expected to become a potential therapeutic approach for patients post-PHx.

**Figure 1:**
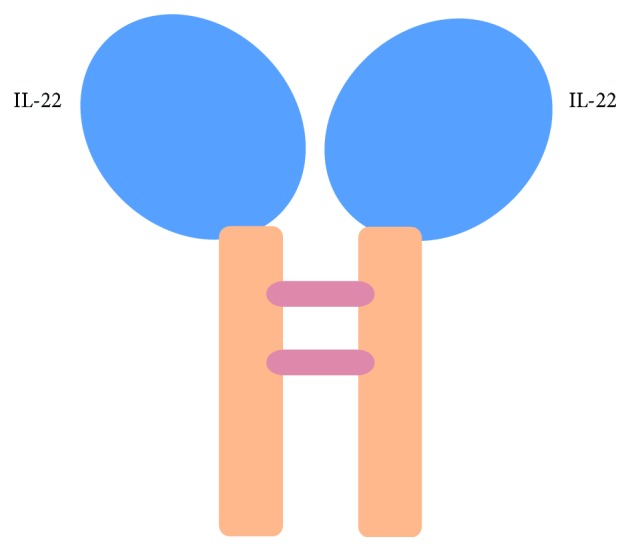
Structure of IL-22-FP. IL-22-FP is manufactured in Chinese hamster ovary (CHO) cells and has a double molecular structure of IL-22 (an IL-22 dimer). As a recombinant fusion protein, IL-22-FP is composed of human IL-22 and human IgG2-Fc.

The majority of clinical patients who receive PHx have a predamaged liver condition caused by various liver diseases and thus obviously decreased liver regeneration ability. However, at present, most of the evidence that supports IL-22 promotion of liver regeneration is from a PHx model without other injuries. In this article, we used ConA and CCl4 to induce liver injury in mice, to investigate the effect of IL-22 on liver regeneration after PHx under these conditions. In addition, currently, almost all the IL-22 used in rodent PHx models is recombinant human interleukin 22 (rh-IL-22). The half-life of rh-IL-22 (expressed in *Escherichia coli*) in animals is less than 2 h, and therefore, repeated administration in a short time is inevitable when rh-IL-22 is used as a therapeutic drug. IL-22-FP, the IL-22 used in this article, is manufactured in Chinese hamster ovary (CHO) cells and has a double molecular structure of IL-22 (an IL-22 dimer, Figure 1). As a recombinant fusion protein, IL-22-FP is composed of human IL-22 and human IgG2-Fc. IL-22-FP has a longer half-life and more easily achieves a stable blood concentration compared to rh-IL-22 [34]. In this article, we are committed to investigating the effects and mechanisms of IL-22-FP on liver protection and regeneration in mice with a predamaged liver condition following PHx, in the hope of providing a new therapeutic means for treatment of clinical patients who undergo liver surgery.

## 2. Materials and Methods

### 2.1. Materials

Male C57BL/6 (8 to 10 wk old) mice used in the experiments were purchased from the Shanghai Lab Animal/Research Center (Shanghai, China). ConA was obtained from Sigma (MO, USA), and CCl4 was obtained from Sinopharm Chemical Reagent Co. Ltd (Shanghai, China). IL-22-FP was provided by GENERON Corporation Ltd. (Shanghai, China); recombinant human interleukin 22 (rh-IL-22) was obtained from Sino Biological Inc. (Beijing, China). Anti-CyclinB1 antibody was purchased from Abcam (Shanghai, China). Other antibodies used in this article, including anti-STAT3, anti-phospho-STAT3 (Tyr^705^), and proliferating cell nuclear antigen (PCNA) antibodies, were purchased from Cell Signaling Technology (Beverly, MA, USA).

### 2.2. Two-Third PHx Model

We performed PHx as previously described [30, 32]. In brief, male C57BL/6 mice were maintained under specific pathogen-free conditions and they had access to water and food freely before each experiment. A midline incision was created, after the mice were anesthetized with ether gas, and the left lateral and median lobes of the liver were freed. Then using a 3-0 silk suture to ligate the blood vessels at the root of the median and left lateral hepatic lobes, the ligated hepatic lobes were resected.

### 2.3. ConA Model

In this model, mice were randomly divided into four groups: the ConA group, PHx group, ConA + PHx group, and ConA + PHx + IL-22-FP group. ConA was administered with a single intravenous injection at 18 *μ*g/g mice body weight, and 4 days later, the PHx was performed. The PHx group mice received identical volumes of phosphate-buffered saline (PBS) in the same way. For the ConA + PHx + IL-22-FP group mice, IL-22-FP was injected intravenously (iv) 30 min before PHx at 0.125 *μ*g/g body weight, and other group animals received an identical volume of PBS in the same manner. Mice were euthanized at different time points (32 h, 40 h, 48 h, and 1 wk) following surgery (each group has 4 mice at each time point), and the liver tissue and serum samples were obtained for subsequent examination.

### 2.4. CCl4 Model

Similar to the ConA model, mice were divided into 4 groups in this model: the CCl4 group, PHx group, CCl4 + PHx group, and CCl4 + PHx + IL-22-FP group. Mice received an intraperitoneal (ip) injection of 10% CCl4 solution (volume of CCl4: volume of olive oil = 1: 9) at 1 *μ*l/g body weight three times a week for 4 wk, while the PHx group mice were administered with only olive oil. As with the ConA model, IL-22-FP was injected intravenously at 0.125 *μ*g/g body weight 30 min before PHx, and other group mice were injected with PBS. Mice were euthanized at the same time points (32 h, 40 h, 48 h, and 1 wk) and in the same way as the ConA model mice (each group has 4 mice at each time point).

### 2.5. Hepatic IRI Model

The hepatic IRI model that we used has been previously described [35, 36]. In brief, male C57BL/6 mice (8–10 wk of age) were anesthetized with chloral hydrate (ip) before a midline incision was created. With full exposure of the structure where it is connected with the portal triad and the median and left liver lobes (including the portal vein, hepatic artery, and bile duct), a vascular atraumatic clamp was used to blocked it for 90 min, and then the clamp was removed, and the liver was reperfused. Mice were euthanized at 6, 24, and 48 h postreperfusion (each group has 4 mice at each time point). Mice were randomly divided into 4 groups: the sham group, IRI group, IRI + rh-IL-22 group, and IRI + IL-22-FP group. The sham group mice received only a midline incision. For IRI + rh-IL-22 group and IRI + IL-22-FP group mice, IL-22 (rh-IL-22 or IL-22-FP) was injected (iv) 30 min before ischemia/reperfusion surgery at 0.125 *μ*g/g body weight, and other mice were treated with PBS.

### 2.6. Liver Weight/Body Weight Ratios (LW/BW)

Mice were euthanized by overdose of anesthesia at 32 h, 40 h, 48 h, and 1 wk after PHx (ConA model and CCl4 model) or at 6, 24, and 48 h postreperfusion (IRI model). The liver and body weights of each mouse were measured, and the liver weight/body weight ratio (LW/BW) was determined to assess the degree of liver regeneration.

### 2.7. Analysis of Liver Injury

Serum aspartate aminotransferase (AST) and alanine aminotransferase (ALT) levels were measured using a standard autobiochemical analyzer (Beckman Coulter AU5800, USA) to assess the degree of liver damage. Liver tissue was fixed with formalin and embedded in paraffin, and the paraffin-embedded liver tissue was cut into sections and stained with hematoxylin and eosin (HE) for histological examination.

### 2.8. BrdU Staining and Analysis

Mice received an intraperitoneal injection of 5-bromo-2′-deoxyuridine (Sigma, Germany) at 50 *μ*g/g body weight 2 h prior to euthanasia. Liver specimens were collected to process for BrdU staining with a BrdU in situ detection kit (BD Bioscience, San Jose, CA, USA). BrdU^+^ hepatocytes are regenerated hepatocytes. The number of BrdU^+^ hepatocytes and total hepatocytes in 4–6 microscope fields (200x) was determined, and the BrdU^+^ hepatocyte/total hepatocyte ratio was calculated; this ratio directly reflected the degree of hepatocyte regeneration.

### 2.9. Western Blots

Proteins were extracted from hepatic tissue for Western blot analysis. The protein concentration was detected with a BCA protein assay kit (Beyotime Biotechnology, Shanghai, China), and the absorbance value of the protein was measured with a microplate reader (BioTek Instruments, USA). The protein samples were separated by SDS-PAGE and transferred to PVDF membranes. The membranes were incubated with primary antibodies including antibodies against PCNA (1 : 1000), CyclinB1 (1 : 2000), p-STAT3 (1 : 1000), and STAT3 (1 : 2000) at 4°C overnight before being incubated with horseradish peroxidase-conjugated secondary antibodies (1 : 5000 dilution) at 4°C for 4 h. Protein bands were visualized with a SuperSignal West Pico Trial Kit (Thermo Scientific, Rockford, USA).

### 2.10. Statistical Analysis

All parametric data are expressed as the mean ± SD. SPSS software was used for statistical analysis. One-way ANOVA was applied to compare differences between groups. *p* < 0.05 was the threshold for statistical significance. The data were analyzed with GraphPad Prism software.

## 3. Results

### 3.1. Enhanced Liver Regeneration after PHx in IL-22-FP-Treated Mice in the ConA Model

**Figure 2:**
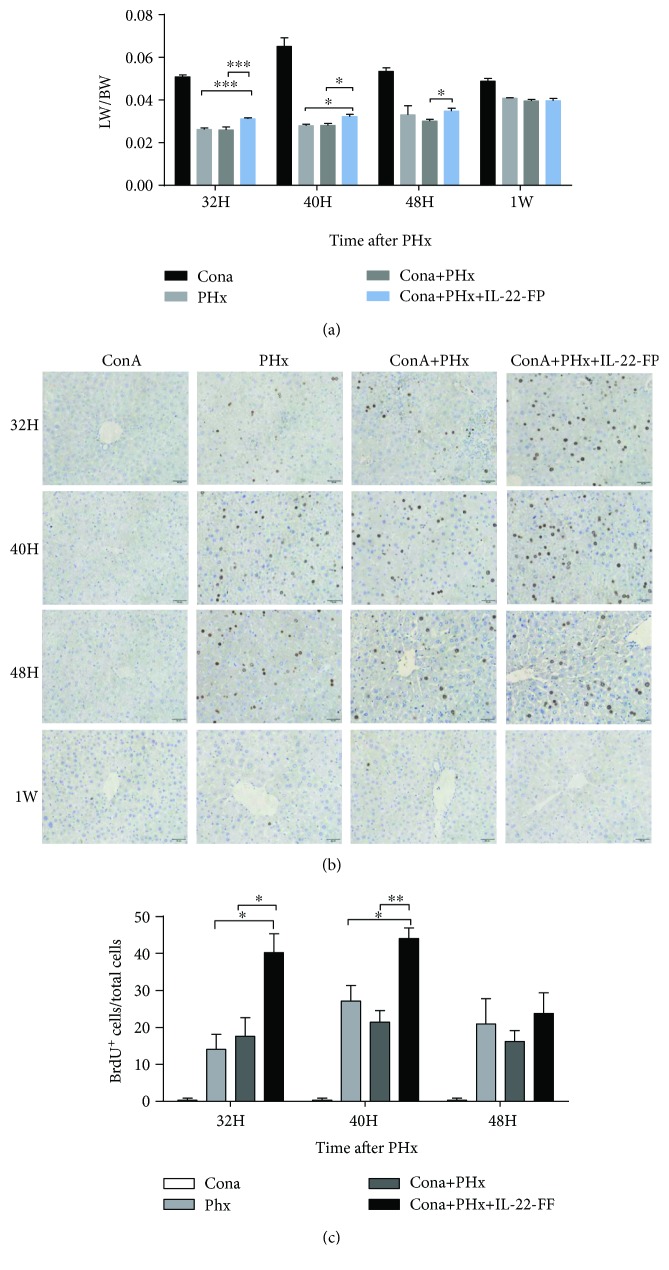
IL-22-FP enhances liver regeneration in a ConA model after PHx. C57BL/6 mice (8 to 10 wk old) were randomly divided into 4 groups: the ConA group, PHx group, ConA + PHx group, and ConA + PHx + IL-22-FP group. Each group of mice was treated as described in Materials and Methods. (a) The liver weight/body weight ratio (LW/BW) of each group at 32 h, 40 h, 48 h, and 1 wk post-PHx; *n* = 4 for each group. (b) Bromodeoxyuridine (BrdU) staining of mouse liver at 32 h, 40 h, 48 h, and 1 wk post-PHx (400x magnification). (c) BrdU^+^ hepatocyte/total hepatocyte ratios of 4 groups of mice at 32, 40, and 48 h post-PHx. Pictures were taken at 400x magnification; *n* = 4 for each group. ^∗^*p* < 0.05, ^∗∗^*p* < 0.01, and ^∗∗∗^*p* < 0.001.

In the ConA model, we divided mice into 4 groups: the ConA group, PHx group, ConA + PHx group, and ConA + PHx + IL-22-FP group, with the specific circumstances described above. After euthanasia, we weighed the liver and body of the mice and LW/BW ratios were calculated to reflect the rate of liver regeneration. The LW/BW ratios at different time points (32 h, 40 h, 48 h, and 1 wk) post-PHx are shown in Figure 2(a). Compared with the ConA + PHx group, the LW/BW ratios of the ConA + PHx + IL-22-FP group were significantly increased at 32 h (^∗∗∗^*p* < 0.001), 40 h (^∗^*p* < 0.05), and 48 h (^∗^*p* < 0.05) after PHx. Similarly, this ratio in the ConA + PHx + IL-22-FP group was also higher than that in the PHx group at 32 h (^∗∗∗^*p* < 0.001) and 40 h (^∗^*p* < 0.05) but this difference was not particularly evident at 48 h. The difference in the ratio of these 3 groups (PHx, ConA + PHx, and ConA + PHx + IL-22-FP) was smaller at 1 wk after PHx.

A portion of the liver tissue was preserved in formalin for BrdU staining (Figure 2(b)). The BrdU^+^ hepatocyte/total hepatocyte ratio was calculated (Figure 2(c)). At 32 h (^∗^*p* < 0.05) and 40 h (^∗∗^*p* < 0.01), this ratio in the ConA + PHx + IL-22-FP group was significantly higher than that in the ConA + PHx group, while this difference at 48 h was small. The number of BrdU^+^ hepatocytes was relatively higher in the ConA + PHx + IL-22-FP group than in the PHx group at 32 h (^∗^*p* < 0.05) and 40 h (^∗^*p* < 0.05) post-PHx.

### 3.2. Hepatoprotective Effect of IL-22-FP after PHx in the ConA Model

**Figure 3:**
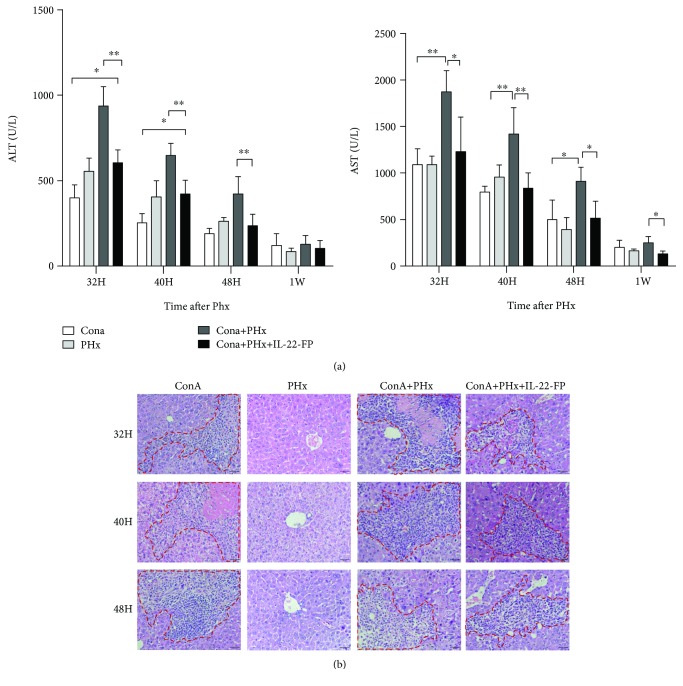
Hepatoprotective effects of IL-22-FP in a ConA model. Mice were grouped and treated as described in Figure 1. (a) At different time points (32 h, 40 h, 48 h, and 1 wk) post-PHx, mice serum samples were collected to detect ALT and AST levels; *n* = 4 for each group. (b) Mice were euthanized at different time points (32 h, 40 h, and 48 h) post-PHx, and liver tissues were stained with hematoxylin and eosin (HE). Pictures were taken at 400x magnification. ^∗^*p* < 0.05 and ^∗∗^*p* < 0.01.

Grouping and modeling were performed as described in Materials and Methods. Serum ALT and AST levels in the ConA model were measured via biochemical analysis. As illustrated in Figure 3(a), the serum ALT levels in the ConA + PHx group were higher than those in the ConA + PHx + IL-22-FP group at 32 h (^∗∗^*p* < 0.01), 40 h (^∗∗^*p* < 0.01), and 48 h (^∗∗^*p* < 0.01) post-PHx. However, this difference was not statistically significant at 1 wk after PHx. Unlike the ALT levels, serum AST levels were significantly greater in the ConA + PHx group than in the ConA + PHx + IL-22-FP group at all observation time points.

Compared with the ConA + PHx + IL-22-FP group mice, the ConA + PHx group mice appeared to suffer from more severe hepatic injury, more extensive tissue necrosis and inflammatory cell infiltration was found in the ConA + PHx group at 32, 40, and 48 h post-PHx (Figure 3(b)).

### 3.3. Enhanced Liver Regeneration after PHx in IL-22-FP-Treated Mice in the CCl4 Model

**Figure 4:**
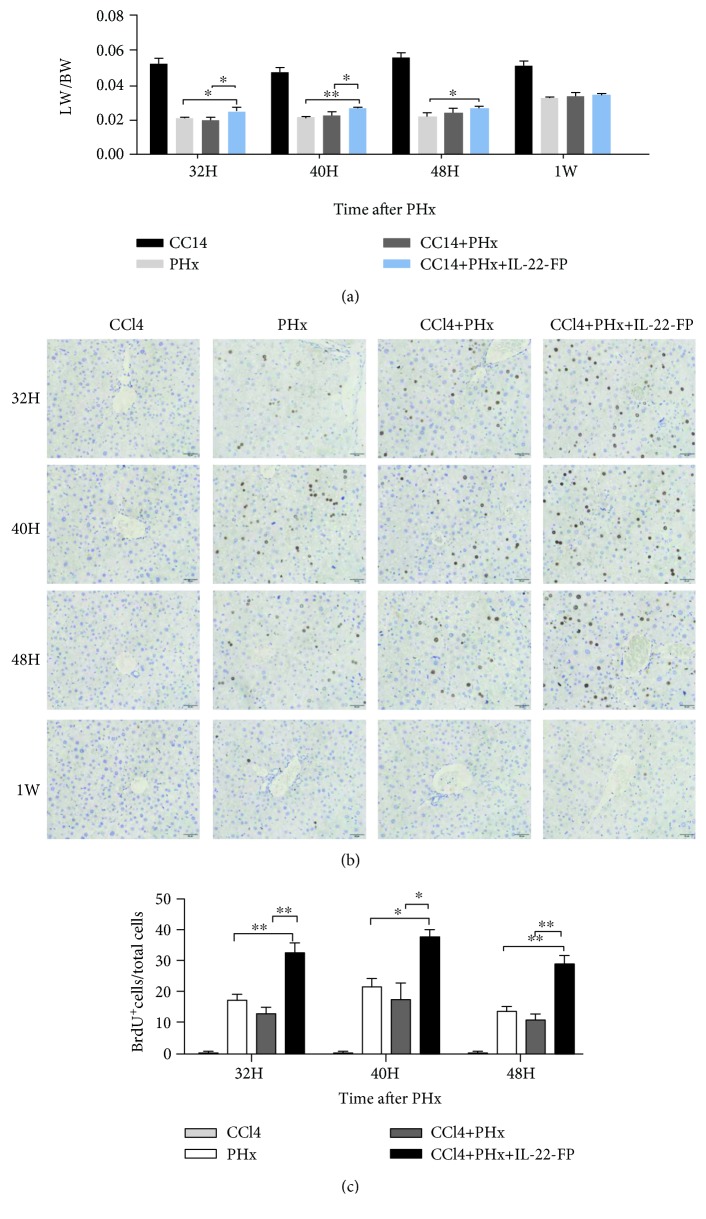
IL-22-FP promotes liver regeneration in a CCl4 model after PHx. Male C57BL/6 mice (8 to 10 wk old) were randomly divided into four groups: the CCl4 group, PHx group, CCl4 + PHx group, and CCl4 + PHx + IL-22-FP group. Mice in different groups were treated as described in Materials and Methods. (a) Liver weight/body weight ratios of different group mice at 32 h, 40 h, 48 h, and 1 wk post-PHx; *n* = 4 for each group. (b) BrdU staining of residual liver in four groups of mice at 32, 40, and 48 h post-PHx (400x magnification). (c) BrdU^+^ hepatocyte/total hepatocyte ratios of four groups at different time points (32, 40, and 48 h) post-PHx. Pictures were taken at 400x magnification; *n* = 4 for each group. ^∗^*p* < 0.05 and ^∗∗^*p* < 0.01.

Mice were randomly divided into 4 groups: the CCl4 group, PHx group, CCl4 + PHx group, and CCl4 + PHx + IL-22-FP group. Different groups of mice were treated as mentioned in Materials and Methods. As shown in Figure 4(a), in the CCl4 + PHx group, the LW/BW ratio of the CCl4 + PHx + IL-22-FP group was increased obviously at 32 h (^∗^*p* < 0.05) and 40 h (^∗^*p* < 0.05) after PHx. At 32 (^∗^*p* < 0.05), 40 (^∗∗^*p* < 0.01), and 48 h (^∗^*p* < 0.05), the LW/BW ratio of the CCl4 + PHx + IL-22-FP group was significantly higher than that of the PHx group. There was no obvious difference in this ratio between the CCl4 + PHx + IL-22-FP group and other groups at 1 wk post-PHx.

Mice were euthanized at 32 h, 40 h, 48 h, and 1 wk after PHx, and the results of BrdU staining in the liver are shown in Figure 4(b). We counted the BrdU^+^ hepatocyte/total hepatocyte ratios at different time points in each group (Figure 4(c)). As expected, in the CCl4 + PHx + IL-22-FP group, the number of BrdU^+^ hepatocytes under the same high-magnification microscope at different time points (32 h, 40 h, and 48 h) was significantly higher than those in the CCl4 + PHx group and the PHx group. As shown in Figure 4(b), almost no BrdU^+^ hepatocytes were found in any of the groups at 1 wk post-PHx.

### 3.4. Hepatoprotective Effect of IL-22-FP in the IRI Model

**Figure 5:**
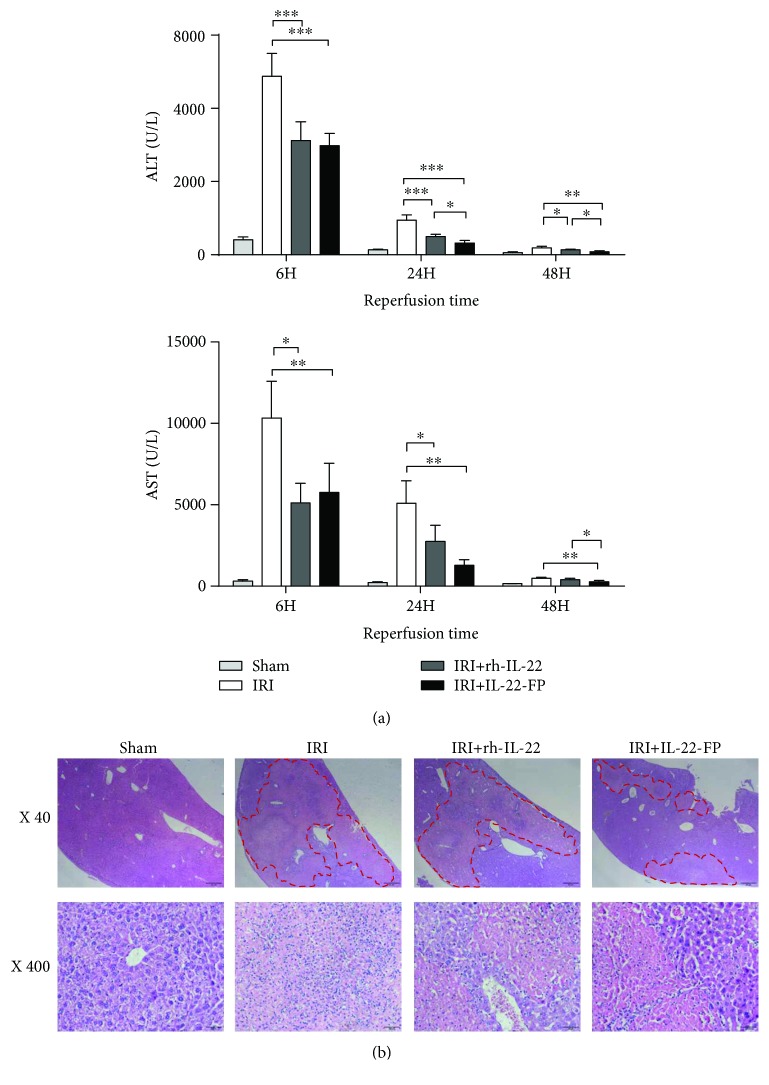
The protective effect of IL-22-FP on the liver in an IRI model. Male C57BL/6 mice (8 to 10 wk old) were randomly divided into 4 groups: the sham group, IRI group, IRI + rh-IL-22 group, and IRI + IL-22-FP group. The different groups of mice were treated as described above. (a) Serum ALT and AST levels in four groups of mice at 6, 24, and 48 h postreperfusion; *n* = 4 for each group. (b) Hematoxylin and eosin staining of liver tissue to display necrosis in liver sections at 48 h postreperfusion (40x or 400x magnification). ^∗^*p* < 0.05 and ^∗∗^*p* < 0.01.

Male C57BL/6 mice were randomly divided into 4 groups: the sham group, IRI group, IRI + rh-IL-22 group, and IRI + IL-22-FP group. The last three groups of mice received 90 min of liver ischemia followed by reperfusion at different time points (6, 24, and 48 h), and the details are described above. As illustrated in Figure 5(a), in mice treated with IL-22 (the IRI + rh-IL-22 group and the IRI + IL-22-FP group), the serum ALT and AST levels at all observation time points were significantly reduced and the effect of IL-22-FP was more obvious than that of rh-IL-22 in reducing the ALT and AST levels at 48 h postreperfusion (^∗^*p* < 0.05).

Liver tissues were stained with hematoxylin and eosin. Necrosis of liver tissue was even worse in the IRI group and the IRI + rh-IL-22 group compared with the IRI + IL-22-FP group at 48 h postreperfusion (Figure 5(b)).

### 3.5. Effects of IL-22-FP on PCNA, CyclinB1, and p-STAT3 Activation after PHx

**Figure 6:**
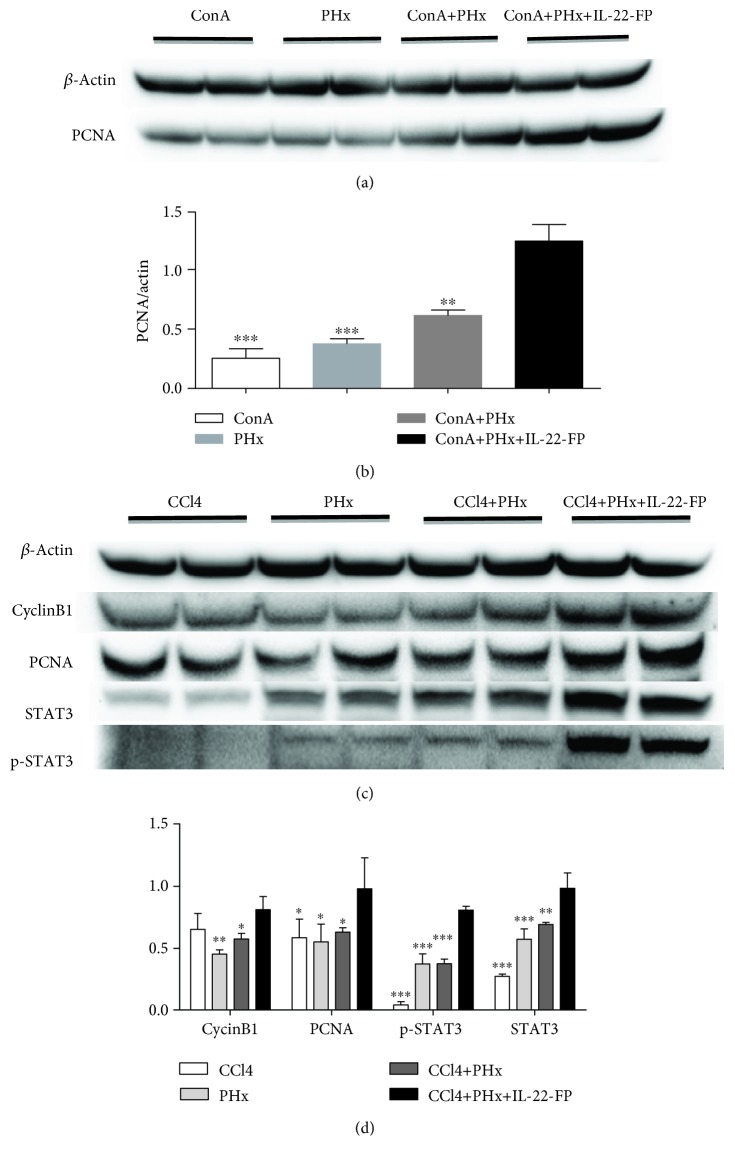
Effects of IL-22-FP on PCNA, CyclinB1, STAT3, and p-STAT3 activation after PHx. After euthanasia, the residual mouse liver was frozen for Western blot analysis. (a, b) PCNA expression was obviously increased in the ConA + PHx + IL-22-FP group compared with that in the other three groups at 40 h post-PHx in the ConA model. ^∗∗^*p* < 0.01 and ^∗∗∗^*p* < 0.001. (c, d) Expression of CyclinB1, PCNA, and p-STAT3 was obviously increased in the CCl4 + PHx + IL-22-FP group compared with that in the other three groups at 40 h post-PHx in the CCl4 model (all statistics were compared with the CCl4 + PHx + IL-22-FP group). ^∗^*p* < 0.05, ^∗∗^*p* < 0.01, and ^∗∗∗^*p* < 0.001.

The expression of PCNA protein in ConA model mice at 40 h post-PHx was detected with Western blotting. As illustrated in Figures 6(a) and 6(b), the activation of PCNA in the ConA + PHx + IL-22-FP group was significantly increased compared with that in the ConA group (^∗∗∗^*p* < 0.001), PHx group (^∗∗∗^*p* < 0.001), and ConA + PHx group (^∗∗^*p* < 0.01). This result indicates that IL-22-FP promoted the expression of PCNA protein after PHx.

As shown in Figures 6(c) and 6(d), the activation of CyclinB1, PCNA, STAT3, and p-STAT3 was obviously increased in the CCl4 + PHx + IL-22-FP group compared with the other groups at 40 h post-PHx in the CCl4 model; it was difficult to observe the expression of p-STAT3 in the CCl4 group.

## 4. Discussion

Partial hepatectomy is still the best available treatment for HCC patients. For a normal liver, the maximum resection rate is 70–75%; when this ratio is exceeded, the patient is likely to experience acute liver failure due to insufficient regeneration of the remnant liver. For patients with liver disease/liver injury pre-PHx, the resection rate is greatly reduced, which severely restricts the use of surgery and the treatment of patients. In this article, we confirmed the hepatoprotective and proregenerative characteristics of IL-22-FP in mice with a predamaged liver condition (caused by ConA/CCl4) and who had undergone PHx. We also confirmed the hepatoprotective effect of IL-22-FP on liver IRI, and this protective effect was stronger than that of rh-IL-22. These properties of IL-22-FP indicate that it is likely a potential therapeutic approach for liver surgery patients.

The hepatoprotective effects of IL-22 in various animal and human liver diseases have been investigated [9, 20, 37, 38]. In 2004, professor Bin Gao's team confirmed that IL-22 mRNA and protein expression was significantly increased in T cell-mediated hepatitis caused by ConA. Blocking IL-22 with neutralizing antibodies worsened the liver damage induced by ConA through reduction in the transduction and activation of the STAT3 pathway, and this type of damage was reduced with administration of recombinant IL-22 [21]. In the same year, this team further confirmed that hydrodynamic gene delivery of IL-22 protects the liver from the damage caused by ConA and CCl4 [20]. In addition, many researchers have confirmed that IL-22 plays a protective role in hepatic injury caused by a variety of toxins such as D-galactosamine (D-GalN) and acetaminophen (APAP) [9, 12, 13, 39]. Unfortunately, at present, the exact mechanism of the hepatoprotective effects of IL-22 is still not very clear. Some researchers propose that this protective role of IL-22 may be related to the expression of IL-22-induced antiapoptotic (such as Bcl-2 and Bcl-xL) and mitogenic (such as c-myc and CyclinD1) proteins after activation of the STAT3 pathway [21]. As the results of our experiments also demonstrate, IL-22-FP protects the liver from serious injury caused by ConA and CCl4 and this has a strong relationship with activation of the STAT3 pathway.

The results of many experiments have confirmed that IL-22 contributes to hepatic regeneration [30–32]. Hepatic IL-22R*α* mRNA and serum IL-22 levels are significantly increased after PHx, and anti-IL-22 antibody administered before hepatectomy can significantly reduce liver regeneration, although treatment with exogenous IL-22 before partial hepatectomy has no effect on hepatocyte proliferation [30]. Further research found that the role of IL-22 in promoting liver regeneration was achieved by increasing hepatocyte proliferation and hepatocyte migration [31]. Moreover, IL-22 promotion of liver regeneration in mice with liver disease (caused by ConA) after PHx has been confirmed [32]. Our findings indicate that IL-22-FP enhances liver regeneration in mice with a predamaged liver condition that have undergone PHx. It is generally accepted that treatment with IL-22 induces activation of STAT3, which then promotes the expression of a variety of cell cycle proteins, eventually promoting hepatocyte proliferation [30–32]. Our research results (Figures 6(a) and 6(c)) suggested that STAT3 was activated significantly in the liver of mice treated with IL-22-FP compared with the other three groups. Activated STAT3 induces the expression of cell cycle proteins (e.g., CyclinB1 and CyclinD1) and increases the expression of PCNA protein significantly in the liver to eventually promotes the proliferation of hepatocytes. These findings suggest that IL-22 is an inducer of STAT3 activation in hepatocytes and activated STAT3 is a contributing factor to the regeneration of hepatocytes. Therefore, IL-22 promotes liver regeneration through the STAT3 pathway.

Liver IRI is one of the major complications in liver resection and liver transplantation [40, 41]. At the onset of reperfusion, an imbalance between nitric oxide levels and endothelin leads to failure of liver microcirculation. Nuclear factor-*κ*B in the liver is activated to promote a combination of proinflammatory cytokines and adhesion molecules, which promotes neutrophil recruitment and oxygen-derived free radical production, further contributing to hepatocyte injury [42, 43]. Paul et al. discovered that IL-22 plays an important role in ischemia-reperfusion-induced liver injury. IL-22 was detected at 24 h postreperfusion, and IL-22R1 expression significantly increased at 6 h postreperfusion in wild-type mice. IL-22 protects the liver against IRI, decreases serum AST levels, alleviates cardinal histological features caused by ischemia-reperfusion, and diminishes leukocyte sequestration [8, 33]. Administration of IL-22-FP significantly decreased serum AST levels at 6, 24, and 48 h postreperfusion (Figure 5(a)). Notably, the application of IL-22-FP led to a more pronounced reduction in AST levels at 48 h postreperfusion compared with rh-IL-22. The hepatoprotective mechanism of IL-22 in IRI is still incompletely understood. One view is that this effect of IL-22 is related to regulation of the inflammatory response [33]. Damaged hepatocytes increased the expression of IL-22-R1 after treatment with IL-22 and IL-22 combined with IL-22-R1, thereby stimulating downstream signaling pathways to promote hepatocyte survival or regeneration [33].

In the liver, only hepatic cells express the IL-22 receptor; immune cells have no IL-22 receptor, and therefore, IL-22 does not target immune cells. Because of this, we have reason to speculate that local or short-term treatment with IL-22 will protect the liver and promote hepatic regeneration without causing excessive inflammation in the liver [44, 45]. Most patients who undergo liver resection have different types of liver disease, and thus, we used ConA and CCl4 to induce liver damage in mice to imitate human liver disease. As our experimental data have shown, IL-22-FP has a significant role in liver protection and enhancing hepatocyte regeneration and these two aspects exactly correspond to the two key problems clinical patients experience after PHx. IL-22-FP ameliorates the liver damage and enhances the proliferation of residual liver cells. In conclusion, IL-22-FP has great potential for treatment of patients with liver disease that have undergone PHx.

## Data Availability

The data used to support the findings of this study have been deposited in the Figshare repository (doi:10.6084/m9.figshare.6287198).

## References

[1] Trivella D. B. B., Ferreira-Júnior J. R., Dumoutier L., Renauld J.-C., Polikarpov I. (2010). Structure and function of interleukin-22 and other members of the interleukin-10 family. *Cellular and Molecular Life Sciences*.

[2] Dudakov J. A., Hanash A. M., van den Brink M. R. M. (2015). Interleukin-22: immunobiology and pathology. *Annual Review of Immunology*.

[3] Sabat R., Ouyang W., Wolk K. (2014). Therapeutic opportunities of the IL-22-IL-22R1 system. *Nature Reviews Drug Discovery*.

[4] Lim C., Savan R. (2014). The role of the IL-22/IL-22R1 axis in cancer. *Cytokine & Growth Factor Reviews*.

[5] Mühl H., Scheiermann P., Bachmann M., Härdle L., Heinrichs A., Pfeilschifter J. (2013). IL-22 in tissue-protective therapy. *British Journal of Pharmacology*.

[6] Parks O. B., Pociask D. A., Hodzic Z., Kolls J. K., Good M. (2016). Interleukin-22 signaling in the regulation of intestinal health and disease. *Frontiers in Cell and Development Biology*.

[7] Park O., Wang H., Weng H. (2011). In vivo consequences of liver-specific interleukin-22 expression in mice: implications for human liver disease progression. *Hepatology*.

[8] Pan C. X., Tang J., Wang X. Y., Wu F. R., Ge J. F., Chen F. H. (2014). Role of interleukin-22 in liver diseases. *Inflammation Research*.

[9] Xing W. W., Zou M. J., Liu S. (2011). Hepatoprotective effects of IL-22 on fulminant hepatic failure induced by d-galactosamine and lipopolysaccharide in mice. *Cytokine*.

[10] Lai R., Xiang X., Mo R. (2015). Protective effect of Th22 cells and intrahepatic IL-22 in drug induced hepatocellular injury. *Journal of Hepatology*.

[11] Zenewicz L. A., Yancopoulos G. D., Valenzuela D. M., Murphy A. J., Karow M., Flavell R. A. (2007). Interleukin-22 but not interleukin-17 provides protection to hepatocytes during acute liver inflammation. *Immunity*.

[12] Scheiermann P., Bachmann M., Goren I., Zwissler B., Pfeilschifter J., Mühl H. (2013). Application of interleukin-22 mediates protection in experimental acetaminophen-induced acute liver injury. *The American Journal of Pathology*.

[13] Ashour T. H. (2014). Therapy with interleukin-22 alleviates hepatic injury and hemostasis dysregulation in rat model of acute liver failure. *Advances in Hematology*.

[14] Ki S. H., Park O., Zheng M. (2010). Interleukin-22 treatment ameliorates alcoholic liver injury in a murine model of chronic-binge ethanol feeding: role of signal transducer and activator of transcription 3. *Hepatology*.

[15] Saalim M., Resham S., Manzoor S. (2016). IL-22: a promising candidate to inhibit viral-induced liver disease progression and hepatocellular carcinoma. *Tumour Biology*.

[16] Lu D. H., Guo X. Y., Qin S. Y. (2015). Interleukin-22 ameliorates liver fibrogenesis by attenuating hepatic stellate cell activation and downregulating the levels of inflammatory cytokines. *World Journal of Gastroenterology*.

[17] Kong X., Feng D., Wang H. (2012). Interleukin-22 induces hepatic stellate cell senescence and restricts liver fibrosis in mice. *Hepatology*.

[18] Kong X., Feng D., Mathews S., Gao B. (2013). Hepatoprotective and anti-fibrotic functions of interleukin-22: therapeutic potential for the treatment of alcoholic liver disease. *Journal of Gastroenterology and Hepatology*.

[19] Jiang R., Tan Z., Deng L. (2011). Interleukin-22 promotes human hepatocellular carcinoma by activation of STAT3. *Hepatology*.

[20] Pan H., Hong F., Radaeva S., Gao B. (2004). Hydrodynamic gene delivery of interleukin-22 protects the mouse liver from concanavalin A-, carbon tetrachloride-, and Fas ligand-induced injury via activation of STAT3. *Cellular & Molecular Immunology*.

[21] Radaeva S., Sun R., Pan H. N., Hong F., Gao B. (2004). Interleukin 22 (IL-22) plays a protective role in T cell-mediated murine hepatitis: IL-22 is a survival factor for hepatocytes via STAT3 activation. *Hepatology*.

[22] Zhang Y., Cobleigh M. A., Lian J.–. Q. (2011). A proinflammatory role for interleukin-22 in the immune response to hepatitis B virus. *Gastroenterology*.

[23] Gao W., Fan Y. C., Zhang J. Y., Zheng M. H. (2013). Emerging role of interleukin 22 in hepatitis B virus infection: a double-edged sword. *Journal of Clinical and Translational Hepatology*.

[24] Zhao J., Zhang Z., Luan Y. (2014). Pathological functions of interleukin-22 in chronic liver inflammation and fibrosis with hepatitis B virus infection by promoting T helper 17 cell recruitment. *Hepatology*.

[25] Dambacher J., Beigel F., Zitzmann K. (2008). The role of interleukin-22 in hepatitis C virus infection. *Cytokine*.

[26] Feng D., Kong X., Weng H. (2012). Interleukin-22 promotes proliferation of liver stem/progenitor cells in mice and patients with chronic hepatitis B virus infection. *Gastroenterology*.

[27] Jia C. (2014). Advances in the regulation of liver regeneration. *Expert Review of Gastroenterology & Hepatology*.

[28] Michalopoulos G. K. (2010). Liver regeneration after partial hepatectomy: critical analysis of mechanistic dilemmas. *The American Journal of Pathology*.

[29] Wang H., Park O., Lafdil F. (2010). Interplay of hepatic and myeloid signal transducer and activator of transcription 3 in facilitating liver regeneration via tempering innate immunity. *Hepatology*.

[30] Ren X., Hu B., Colletti L. M. (2010). IL-22 is involved in liver regeneration after hepatectomy. *American Journal of Physiology. Gastrointestinal and Liver Physiology*.

[31] Brand S., Dambacher J., Beigel F. (2007). IL-22-mediated liver cell regeneration is abrogated by SOCS-1/3 overexpression in vitro. *American Journal of Physiology. Gastrointestinal and Liver Physiology*.

[32] Zhang Y. M., Liu Z. R., Cui Z. L. (2016). Interleukin-22 contributes to liver regeneration in mice with concanavalin A-induced hepatitis after hepatectomy. *World Journal of Gastroenterology*.

[33] Chestovich P. J., Uchida Y., Chang W. (2012). Interleukin-22: implications for liver ischemia-reperfusion injury. *Transplantation*.

[34] Tang K. Y., Lickliter J., Huang Z. H. (2018). Safety, pharmacokinetics, and biomarkers of F-652, a recombinant human interleukin-22 dimer, in healthy subjects. *Cellular & Molecular Immunology*.

[35] Eggenhofer E., Sabet-Rashedi M., Lantow M. (2016). ROR*γ*t^+^ IL-22-producing NKp46^+^ cells protect from hepatic ischemia reperfusion injury in mice. *Journal of Hepatology*.

[36] Yu J., Feng Z., Tan L., Pu L., Kong L. (2016). Interleukin-11 protects mouse liver from warm ischemia/reperfusion (WI/Rp) injury. *Clinics and Research in Hepatology and Gastroenterology*.

[37] Abe H., Kimura A., Tsuruta S. (2014). Aryl hydrocarbon receptor plays protective roles in ConA-induced hepatic injury by both suppressing IFN-*γ* expression and inducing IL-22. *International Immunology*.

[38] Wahl C., Wegenka U. M., Leithauser F., Schirmbeck R., Reimann J. (2009). IL-22-dependent attenuation of T cell-dependent (ConA) hepatitis in herpes virus entry mediator deficiency. *The Journal of Immunology*.

[39] Feng D., Wang Y., Wang H. (2014). Acute and chronic effects of IL-22 on acetaminophen-induced liver injury. *Journal of Immunology*.

[40] Chen Y., Lv L., Pi H. (2016). Dihydromyricetin protects against liver ischemia/reperfusion induced apoptosis via activation of FOXO3a-mediated autophagy. *Oncotarget*.

[41] Pasut G., Panisello A., Folch-Puy E. (2016). Polyethylene glycols: an effective strategy for limiting liver ischemia reperfusion injury. *World Journal of Gastroenterology*.

[42] Serracino-Inglott F., Habib N. A., Mathie R. T. (2001). Hepatic ischemia-reperfusion injury. *American Journal of Surgery*.

[43] Weigand K., Brost S., Steinebrunner N., Büchler M., Schemmer P., Müller M. (2012). Ischemia/reperfusion injury in liver surgery and transplantation: pathophysiology. *HPB Surgery*.

[44] Gao B. (2015). Interplay of interleukin-22 and its binding protein in controlling liver scarring. *Hepatology*.

[45] Park O., Ki S. H., Xu M. (2015). Biologically active, high levels of interleukin-22 inhibit hepatic gluconeogenesis but do not affect obesity and its metabolic consequences. *Cell & Bioscience*.

